# Tailored FcγR blockade enhances immune checkpoint therapy and overcomes resistance

**DOI:** 10.1186/s13046-026-03785-5

**Published:** 2026-07-25

**Authors:** Robert J. Oldham, Linda Mårtensson, Monika Semmrich, Niyaz Yoosuf, Petra Holmkvist, Lara V. Graham, Martin C. Taylor, Kirstie L. S. Cleary, Mona Yazdani, Josephine F. Buckingham, Ali Roghanian, Ingrid Karlsson, Stephen A. Beers, Ingrid Teige, Mark S. Cragg, Björn Frendéus

**Affiliations:** 1 Antibody and Vaccine Group, School of Cancer Sciences, University of Southampton, Southampton, UK; 2 BioInvent, Lund, Sweden

**Keywords:** Fc gamma receptors, Immune checkpoint blockade, Antibody Immunotherapy, Drug Resistance

## Abstract

**Background:**

Fc-gamma receptors (FcγRs) regulate IgG antibody activity, and Fc-engineering is a proven method to improve the efficacy of tumor-targeting antibodies. Here, we explore tailored FcγR blockade to enhance the therapeutic efficacy and tolerability of immune checkpoint-blocking (ICB) antibodies.

**Methods:**

Mechanistically matched murine surrogate and human lead FcγR-blocking and immune checkpoint-blocking antibodies were used to study whether tailored FcγR-blockade, targeting FcγRIIB selectively or all FcγRs, can enhance the efficacy and overcome resistance to immune checkpoint therapy in vivo and in vitro. Mechanistic studies were performed with clinical reagents, including ipilimumab, nivolumab, pembrolizumab, and human FcγRIIB-selective (BI-1607) and pan-FcγR-blocking (BI-1206) antibodies, using human cells and transgenic animals with clinically relevant expression of immune checkpoint receptors.

**Results:**

We demonstrate that FcγRIIB-selective and pan-FcγR-blocking antibodies increase the in vivo efficacy of αCTLA-4 and αPD-1 antibodies, respectively. FcγRIIB-selective antibody enhancement of αCTLA-4 was associated with increased intratumoral Treg depletion, myeloid reprogramming, interferon-γ and CXCL10-induction, and increased activated effector CD8^+^ T cells, correlating with higher activating-to-inhibitory (A:I) FcγR engagement ratios. Conversely, pan-FcγR blockade protected αPD-1-coated T cells from macrophage phagocytosis, increasing intratumoral activated CD8^+^ T cells by decreasing activating and inhibitory FcγRs.

**Conclusions:**

Our studies provide in vivo proof of concept that tailored FcγR blockade enhances immune checkpoint therapy and overcomes resistance through mechanistically distinct pathways. Clinical trials with tailored human FcγRIIB-blocking antibodies are ongoing.

**Graphical Abstract:**

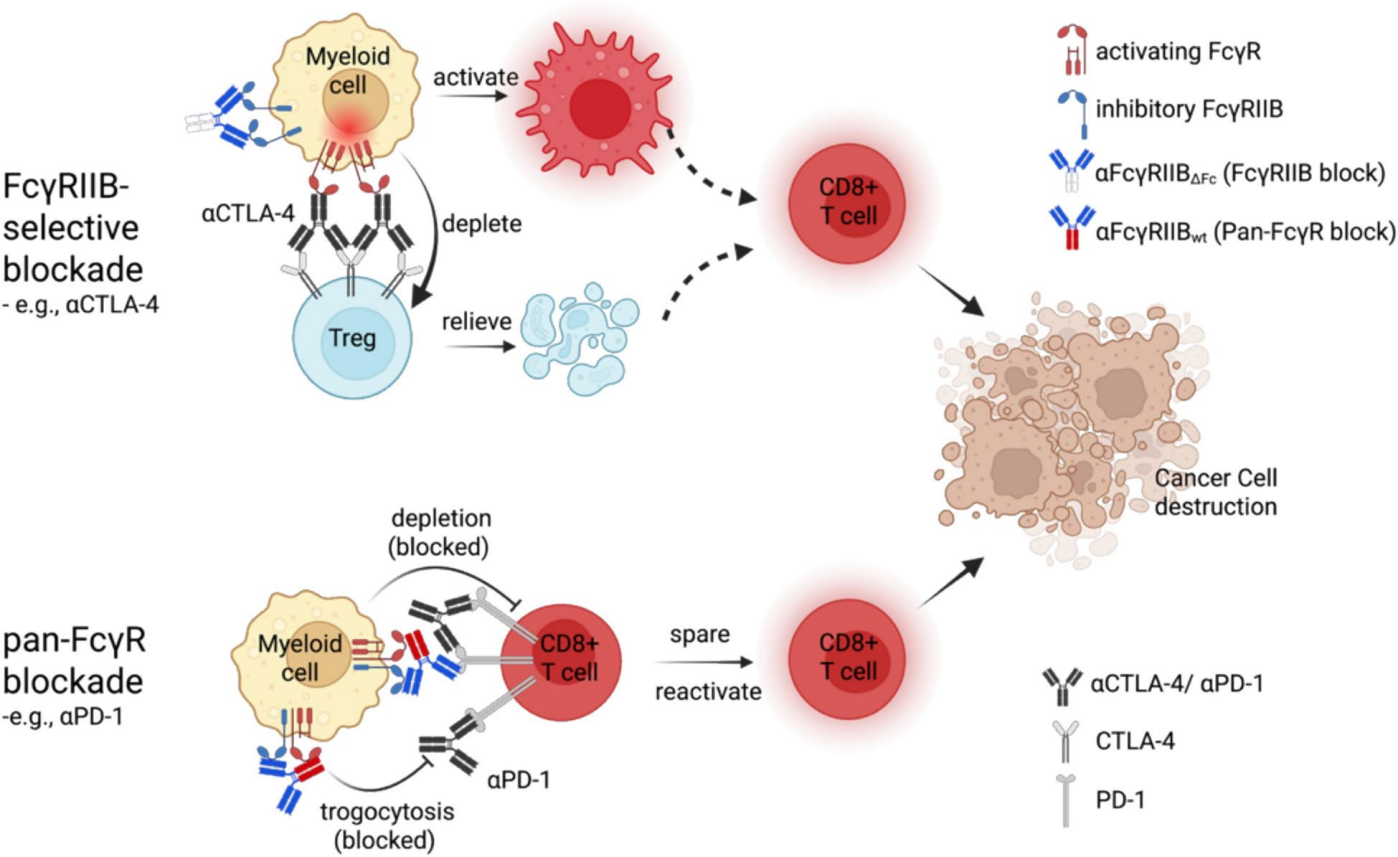

- FcγRIIB-selective blockade enhances αCTLA-4-mediated Treg depletion, myeloid reprogramming, and effector CD8^+^ T cell activation by increasing the activating-to-inhibitory (A:I) FcγR engagement ratio.
- Conversely, pan-FcγR blockade protects αPD-1-coated T cells from macrophage phagocytosis and from αPD-1 removal via trogocytosis, thereby increasing intratumoral activated CD8^+^ T cells by decreasing activating and inhibitory FcγRs.

**Supplementary Information:**

The online version contains supplementary material available at 10.1186/s13046-026-03785-5.

## Introduction

Immune checkpoint blockade (ICB) with antibodies targeting PD-1, PD-L1, or CTLA-4 has transformed cancer survival and is used to treat more than forty cancer types [1]. Despite this, many patients fail to respond or develop resistance during therapy [2]. Combining αPD-1 and αCTLA-4 increases efficacy, but dose-dependent toxicity limits the use of available αCTLA-4 antibody regimens [3]. Identifying and overcoming mechanisms that mediate resistance to αPD-(L)1 and developing means to deliver safer, efficacious αCTLA-4-based therapies could improve survival and bring the clinical benefits of ICB to currently non-responding cancer types and patients.

Fc gamma receptors (FcγR) regulate IgG antibody activity, and their importance for tumor-targeting antibodies is well documented [4–6]. Several activating FcγRs (FcγRI, IIA, and IIIa) and a single inhibitory FcγR (FcγRIIB) collectively regulate FcγR^+^ immune effector cell-mediated responses during antibody therapy [4, 6]. A high ratio of activating to inhibitory FcγRs promotes effector cell activation and downstream cellular events, including the depletion of antibody-coated target cells [7]. Consistent with the positive and negative regulation of IgG-induced effector cell responses by activating and inhibitory FcγRs, we previously demonstrated the therapeutic potential of blocking the inhibitory FcγRIIB to improve antibody therapy. Antagonistic FcγRIIB antibodies enhanced the activity of rituximab and trastuzumab and overcame rituximab resistance in vivo [8], with clinical investigation ongoing (NCT03571568).

Emerging data suggest that FcγRs also influence the effectiveness of immune-modulatory antibodies, including those directed against CTLA-4 and PD-(L)1 (reviewed in [9, 10]). These antibodies were created to block inhibitory signals in CD8^+^ T cells, effectively releasing the brakes on the immune system to eliminate cancer cells. This process does not rely on a role for FcγRs. Surprisingly, as seen with tumor-directed antibodies, Vargas et al. reported that FcγRs play a part in the therapeutic action of the CTLA-4-specific antibody ipilimumab [11]. Dahan et al. discovered that αPD-1 and αPD-L1 antibodies have opposing requirements for FcγR engagement in preclinical models [12]. Since then, we and others have found that FcγRs may enhance the effectiveness of αCTLA-4 and other immune-modulatory antibodies through various mechanisms, including Treg depletion [11, 13], improved T cell–APC immune synapse formation [14–17], and reprogramming tumor-associated macrophages [16, 17]. On the other hand, some studies indicate a harmful role for FcγRs in the efficacy of αPD-1 antibodies, with conflicting reports on the roles of activating and inhibitory FcγRs [12, 18–20]. Importantly, how FcγRs regulate the activities of individual ICB antibodies remains only partly understood, which limits rational optimization. Additionally, while Fc-engineering is a proven approach to boost therapeutic antibody efficacy, creating new Fc-modified antibodies from existing approved drugs with refined FcγR engagement would be a long and expensive process. Therefore, we have focused on an alternative approach: combining current, approved immune checkpoint therapies with specific FcγR blockade.

Here, we examined the impact of FcγR blockade on the therapeutic effectiveness of αCTLA-4 and αPD-1 antibodies using FcγRIIB-specific antibodies with Fc constant domains capable of binding multiple FcγRs (hIgG1 ‘BI-1206’ or mIgG2a) or genetically engineered to silence FcγR engagement (hIgG1N297Q ‘BI-1607’ or mIgG1N297A). We further characterize these reagents and offer a mechanistic explanation for their different abilities to enhance the therapeutic activity of αCTLA-4 and αPD-1 antibodies. To maximize αCTLA-4 antibody effectiveness, selective blockade of FcγRIIB with Fc-impaired αFcγRIIB was necessary. Conversely, optimal αPD-1 activity required blocking all FcγRs with Fc-competent αFcγRIIB. Our findings suggest strategies to improve clinically approved αPD-1 antibodies, like pembrolizumab and nivolumab, and to support safe and effective dual αPD-1 and αCTLA-4 immune checkpoint blockade (ICB). Both approaches are currently under clinical trial investigation, with the potential to expand treatment options to resistant cancers.

## Materials and methods

### Generation of antibodies targeting FcγRII, PD-1, PD-L1 and TIGIT

αPD-1 (clone RMP1-14), αPD-L1 (clone MPDL3280A, atezolizumab), and αTIGIT (clone 10A07) were cloned onto different mouse IgG-backbones. The antibodies were expressed in suspension-adapted HEK 293 EBNA cells (Thermo Fisher Scientific) and purified from the cell supernatants on Mabselect (GE Healthcare). α-FcγRIIb (clone AT130-2) was either produced as described above or produced in ExpiCHO-S cells using the ExpiCHO transient expression system (ThermoFisher) and purified by protein A affinity chromatography using a HiTrap MAbSelect SuRe protein A column (Cytiva), followed by size exclusion chromatography (SEC) using a HiLoad Superdex 200 pg 16/600 SEC column (Cytiva), if required. All antibodies were determined to be endotoxin low (< 1 EU/mg) using an Endosafe Portable Test System device (Charles River Laboratories) and aggregate-free (< 1%) using high-performance liquid chromatography (HPLC). Clinically, relevant α-human PD-1 antibodies (nivolumab and pembrolizumab) were obtained from the University Hospital Southampton pharmacy.

### Surface plasmon resonance analysis

Binding of AT130-2 mIgG2a and mIgG1 N297A to mouse FcγR was assessed by Surface Plasmon Resonance using a Biacore T200 instrument (Cytiva). Antibodies were immobilized on a CM5 chip (Cytiva) via amine coupling. Recombinant mouse FcγR (R&D Systems) were diluted in HBS-EP + (Cytiva). A 5-fold dilution series was used starting at 100 nM for FcγRI and 1000 nM for all other FcγR, these were flowed over the immobilised antibody at a rate of 30 µl/ml for 2 min (5 min for FcγRI) followed by 5-min dissociation. The chip was regenerated between samples with glycerol pH1.5. Samples were analysed in Biacore Evaluation software (Cytiva) with background binding to the control flow cell removed.

### Mice

Six to eight weeks-old (weight 17-20 grams) female C57BL/6J (RRID: IMSR_RJ:C57BL-6JRJ, RRID: IMSR_CRL:027) or BALB/c mice were either purchased from Janvier (Saint-Berthevin, France) and maintained at BioInvent local facilities or purchased from Charles River (Kent, UK) and bred and maintained in UK Home Office approved local facilities. Humanized CTLA-4 mice (B-hCTLA-4) were purchased from Biocytogen (Shanghai, China) and maintained at BioInvent local facilities.

For in vivo experiments performed at BioInvent, all mice were housed and maintained in accordance with Swedish Laboratory Animal Care guidelines and were performed in an animal care-accredited facility. All experimental animal studies were conducted under the approval of the Swedish Ethical Committee (approval numbers 5.8.18–17196/2018 and 5.8.18–19686/2022) and carried out in accordance with the Federation of Laboratory Animal Science Associations (FELASA) guidelines. For in vivo experiments performed at University of Southampton, all mice were housed in IVC units and maintained in accordance with the Animal (Scientific Procedures) Act 1986 and experiments were performed under approved project licenses (P4D9C89EA and PP5396109), and ethical review by local University of Southampton animal welfare committee (approval numbers 69840, 65400).

### In vivo mouse tumor models

The CT26 (CRL-2638) and B16 (CRL-6475) murine cancer cell lines were obtained from ATCC. The MC38 cell line was kindly gifted by Dr Sjef Verbeek. Cells were cultured according to providers’ instructions and logarithmic growth phase of cells was ensured before harvesting cells for engraftment. A total of 0.5 × 10^6^ or 1 × 10^6^ CT26, B16 or MC38 cells were inoculated subcutaneously into the flank of each mouse. All tumor cells were inoculated in 100 µl of PBS. Mice bearing CT26 or MC38 tumors of 40–200 mm^3^ (depending on model and setting) were randomized into treatment groups based on tumor size. B16 mice were treated after 4 days. Mice were treated with the following antibodies or antibody combinations. Anti-mouse PD-1 (29F.1A12, rat IgG2a, RRID: AB_2687796, BioXcell or Assay Genie, RMPI-1 mIgG2a, mIgG1 and mIgG1-N297A or RMPI-1 mIgG2a-AF647 or mIgG1-N297A-AF647), anti-mouse CTLA-4 (9H10, hamster IgG, RRID: AB_10949609, BioXcell), anti-FcγRII (AT130-2 mIgG2a and mIgG1-N297A), anti-human CTLA-4 (ipilimumab, human IgG1, Apoteket), anti-mouse PD-L1 (mIgG1-atezolizumab), anti-mouse TIGIT (10A7 mIgG2b or mIgG2a) and/or control antibodies mIgG2a, mIgG1 or hIgG1 isotype. Antibodies were administered intraperitoneally in a volume of 200 µl.

For tumor growth inhibition studies, mice were treated 3 times (2–3 days apart). Animals were continuously monitored, and mice were euthanized when any of the following end points were met: study termination, tumor burden ≥ 2,000 mm^3^, excessive tumor ulceration, or moribund appearance. Tumor burden was measured using calipers, and tumor volumes were calculated using the modified ellipsoid formula 0.5 × (length × width^2^).

For rechallenge studies, cured mice were inoculated subcutaneously into the opposite flank from the cured primary tumor with 5 × 10^6^ MC38 cells. This was done within a minimum of 6 weeks post complete cure.

For tumor-infiltrating immune cell analysis (by single cell RNA sequencing or by flow cytometry) mice were euthanized 1 h—24 h after the first or third treatment (depending on experimental set-up) and tumors were collected.

Tumors were diced and incubated with either 1 Wunsch unit/ml Liberase TL (Roche) in PBS at 37 °C at 250 rpm for 20 min or in a solution of 50% Liberase TL (Roche) and 50% DNAse (Roche) for 3 × 5 min with slight vortexing in between at 37 °C, 5% CO_2_. The digestion reaction was quenched with 5 mL of RPMI (containing 10% FCS) and homogenized into a single cell suspension through a 100 μm cell strainer. Lymph nodes and spleen did not undergo enzymatic digestion but were homogenized into a single cell suspension through a 100 μm cell strainer. Red blood cells in the spleen were lysed using Lysis Buffer (Gibco or Bio-Rad).

### Tumor-infiltrating immune cell analysis using single cell RNA sequencing

#### Cell preparation

Tumors harvested as indicated were enzymatically digested as described. Viable lymphocytes were isolated using gradients (Lympholyte-M Separation Media, Cedarlane). Tumor lymphocytes were stained with viability dye and CD45 antibody (see antibody Table S1) and viable CD45^+^ cells were sorted using a FACS Aria Fusion (BD). Cells were counted (NucleoCounter NC-202, ChemoMetec) and using 4% paraformaldehyde (PFA) were fixed for 20 h at 4 °C, followed by long-term storage at –80 °C as instructed by ‘10 × Genomics’ protocol. Samples were further processed at the Center for Translational Genomics (CTG) at the medical faculty at Lund University, Sweden. In short, prior to library preparation, samples were thawed, washed, and hybridized with barcoded probe sets according to the Chromium Next-GEM Flex protocol (10 × Genomics). Approximately 10^4^ cells per sample were targeted for capture, and the pooled samples were loaded onto a Chromium X instrument using a Chip Q. The generated libraries were sequenced on an Illumina NovaSeq 6000, targeting a sequencing depth of 2 × 10^4^ reads per cell.

#### Single cell RNA analyses

The scRNA-seq reads were aligned to the mouse transcriptome and a gene expression matrix was generated using the Cell Ranger pipeline using default settings. Using the R Seurat package (v4.4.0), the output files from cellranger were converted to a Seurat object for downstream analysis [21]. The R package Doubletfinder (v2.0.3) was applied with default parameters to find and remove cell doublets in each sample individually [22]. The cell cycle phase was controlled with the Seurat *CellCycleScoring* function using cell cycle related genes. The number of detected genes, the number of detected UMIs, house-keeping gene expression and the percentage of mitochondrial gene expression were used to remove low-quality cells. The *SCTransform* function was performed to normalize samples and applied separately for each dataset after finding the top 3000 feature genes using *SelectIntegrationFeatures* function. Then we applied the functions *PrepSCTIntegration*, *FindIntegrationAnchors* to find integration anchors and *IntegrateData* (normalization.method = “SCT”) to complete data integration.

We ran principal component analysis on the gene space using *RunPCA* and clustering was performed based on the shared nearest neighbor between cells (*FindNeighbors*) and graph-based clustering (*FindClusters*). We used uniform manifold approximation and project (UMAP) with the function *RunUMAP* with the same number of principal components used for embedding for the visualization of clusters. For the UMAP based clustering, we used the top 30 calculated PC dimensions and a resolution of 0.8, eventually identified 25 distinct immune cell subpopulations. To identify marker genes, we applied *FindAllMarkers* function in Seurat (one-sided Wilcoxon rank sum test) with *p*-value adjusted for multiple testing using the Bonferroni correction. Using well curated cell specific marker genes and based on prior experience, we annotated the immune cell subpopulations. For cell-type specific sub-clustering, we integrated the data across samples and performed *SCTransform* approach as mentioned above with parameters ranging from 20–30 PCs and UMAP resolution ranging between 0.2–0.7 with final parameters selected to generate consistent visualizations. *AverageExpression* function in Seurat was used to measure the average expression of gene markers and z-scores per genes were calculated for visualization with the pheatmap package. The R package LSD (https://cran.r-project.org/web/packages/LSD/index.html) was used to visualize density plots of cells using the UMAP coordinates of cells from each condition. All computational analyses were performed using publicly available software packages, as described above (Materials and methods). No custom code or novel computational algorithms were developed for this study.

### Immune cell analysis using flow cytometry

#### Characterization of immune cell contents in tumors across different mouse models

WT mice were inoculated with either CT26, (BALB/c) or MC38 (C57BL/6J) tumor cells and tumors were monitored for growth with calipers. Mice were treated as described for therapy experiments above. When CT26 tumors reached approx. 600–700 mm^3^ or MC38 tumors reached approx. 1300–1400 mm^3^ in area, mice were sacrificed, and samples were processed as described above. Immune cell populations were identified using fluorochrome labeled antibodies (see table S1). After cell surface staining, cells were permeabilised using the Foxp3/Transcription Factor staining buffer set (Invitrogen) before staining for intracellular proteins. Samples were acquired on an LSR Fortessa × 20 (Beckton Dickenson) flow cytometer and analysed using FlowJo software (RRID:SCR_000410).

#### PD-1 expression on human tissue

Cryopreserved, dissociated tumor cells were purchased from Discovery Life Sciences. Material from 8 different indications with 1–5 tumors per indication were included in the analyses and stained with antibodies against CD45, CD3, CD8 and PD-1 (see table S1). αPD-1 (clone 1D6E10; pembrolizumab) was produced in-house and labeled with Alexa Fluor 647. PD-1 expression on CD8^+^ T cells was analysed by flow cytometry, samples were acquired on a FACSAria Fusion (BD) flow cytometer. Dead cells were excluded based on positive staining for the fixable viability dye eFluor 780 (Thermo Fisher Scientific).

### Cytokine analysis in murine tumors and serum

For protein analysis, MC38 tumors were harvested day 7 post first treatment, weighed and placed into homogenization liquid (Pierce RIPA buffer (Merck) and 1:100 Protease inhibitor cocktail (Merck)), 3 μ1/mg tumor. Tumors were homogenized (GentleMACS, Miltenyi) and subsequently centrifuged (10 min at 10,000 × g). Supernatant was stored at −80 °C degrees until analysis.

Blood was drawn from vena saphena (BD Vacutainer Heparin tubes) from MC38 tumor-bearing mice treated in total 3 times with 10 mg/kg of aFcγRII_ΔFc_, alone or in combination with 1 mg/kg of ipilimumab (day 1, 4 and 8). Blood was drawn from 5 mice at each time-point (24, 72 and 168 h).

Levels of 5 cytokines (TNF-α, IFN-γ, CCL22, IL-β and IL-6,) were assessed in serum and tumor using a U-plex assay (MSD) and read by a QuickPlex SQ 120 Reader (MSD). Data were analyzed using Discovery Workbench v.4.0.

### Generation of human-PD-1 expressing Jurkat cells

The full-length human PD-1 gene was cloned into pCDNA3 and nucleofected into Jurkat cells using the Amaxa cell line Nucleofector kit V and program X-001 on the nucleofector II device. Positive cells were selected with 1 mg/ml geneticin (Life Technologies) for 2 weeks before being stained for PD-1 expression and FACS sorted to obtain pure populations of differing expression levels (FACSAriaII, BD Biosciences). Cells were maintained in complete RPMI + 1 mg/ml geneticin.

### Generation of monocyte derived macrophages (MDM) and in vivo activated T cells

Leukocyte cones were obtained from Southampton NHS Blood and Transplant service. Use was approved by the University of Southampton Faculty of Medicine Ethics Committee and East of Scotland Research Ethics Service, Research ethical committee reference number 16/ES/004. Peripheral blood mononuclear cells were isolated by density gradient centrifugation (Lymphoprep, Stem Cell Technologies). MDM were then isolated via adherence or CD14^+^ isolation. For adherence, 1 × 10^7^ PBMCs in 2 ml RPMI + 1% human AB serum were incubated in 6-well plates for 2 h at 37 °C + 5% CO_2._ Non-adherent cells were collected and used to generate activated T cells. Plates were washed with PBS and MDM differentiated over 7 days in complete RPMI + 100 ng/ml M-CSF (in-house). Non-adherent PBMCs were resuspended at 1 × 10^6^ cells/ml in cRPMI with the addition of Dynabeads human T-activator CD3/CD28 at 0.4 × 10^6^ beads/ml + 30 IU/ml IL-2 (Peprotech). Beads and cytokine were replaced twice over 8 days with beads removed using a magnet.

For phagocytosis with subsequent FcγR staining, CD14^+^ cells were isolated by positive selection using CD14^+^ MicroBeads (Miltenyi Biotec). Cells were differentiated in a 96 well plate over 7 days in complete RPMI + 100 ng/ml M-CSF.

### Phagocytosis assay

Adherence isolated MDM were harvested and re-plated at 5 × 10^4^ macrophages/well in a 96 well plate in 100 μl cRPMI + 100 ng/ml M-CSF and incubated overnight at 37 °C with 5% CO_2_. For phagocytosis assays macrophages were treated with αFcγRIIB at 20 μg/ml (2 × final concentration) in 50 μl cRPMI for 30–45 min or 10 µg/ml overnight (FcγR detection). Meanwhile, target cells (Jurkat-hPD1 or activated T cells) were labelled with 5 μM CFSE before being resuspended at 5 × 10^6^/ml in cRPMI and opsonized with αPD-1 at 2 × final concentration for 15 min at room temperature. 50 μl target cell suspension (5:1 target:effector ratio) was added per well of MDM and incubated for 1 h at 37 °C. Macrophages were stained with αCD14 APC/BV421 (Biolegend/BD Biosciences, table S1). For FcγR staining, cells were additionally stained with αFcγR specific antibodies. Phagocytosis assessed by flow cytometry as the proportion of CD14^+^CFSE^+^ cells as a total of CD14^+^ macrophages using a FACS Canto II (BD Biosciences) or Cytoflex (Beckman Coulter).

For imaging, CD14^+^CFSE^+^ cells were FACs sorted onto Superfrost slides (ThermoFisher) using a FACSMelody (BD Biosciences). Slides were fixed with 4% paraformaldehyde for 10 min and mounted with VECTASHIELD hard set with DAPI. Slides were imaged with an Olympus IX81 inverted microscope (Xcellence pro software) and images processed in ImageJ (Fiji).

### Trogocytosis assay

MDM were differentiated over 7 days and plated in 96 well plates as described. 2 h after plating 80 μM dynasore dynamin inhibitor (Abcam) was added and MDM incubated at 37 °C for 24h. FcγRIIB antibodies were added at 20 μg/ml (2 × final concentration) and incubated for 30–60 min. Meanwhile, human PD1 Jurkat cells were opsonized with 2 or 20 μg/ml (2 × final concentration) Alexa Fluor488 labelled nivolumab. Opsonised cells were added to MDM at a 5:1 target:effector ratio and incubated at 37 °C for 1 h. Jurkat cells and CD14^+^ macrophages were analysed by flow cytometry (FACS Canto II, BD Biosciences) for loss of labelled nivolumab from the Jurkat cells and uptake by MDM.

### Statistical analysis

Statistical significance was performed using GraphPad Prism software (GraphPad, RRID:SCR_002798). For comparisons between two groups with normal distribution, Student’s T test was used. If the data points in each group originated from the same donor, paired Student’s T test was used. For comparisons regarding one parameter using three or more groups with normal distribution, one-way ANOVA test was used. If the data points in each group originated from the same donor, Repeated Measures ANOVA test was used. For comparisons regarding one parameter but over several e.g. cell types using three or more groups with normal distribution, two-way ANOVA test using Tukey’s adjustment for multiple comparisons was used. For survival curves, Log-Rank Mantel Cox test was used. Number of data points, number of experiments, graphic interpretation (mean and SD etc.), units, statistical test used and *p*-value limits have been stated in figure legends. Individual data points were shown when possible. ScRNA data processing has been described above, and no statistical analysis was performed in this data set.

## Results

### Generation of mFcγRII-blocking surrogate antibodies

We previously developed human antibodies that effectively block the inhibitory hFcγRIIB [8]. Two antibody variants derived from the hFcγRIIB-specific antibody 6G11 were generated: a hIgG1 antibody (BI-1206) with a wild-type Fc domain that binds both activating and inhibitory FcγRs (‘αFcγRIIB_wt_’), and a hIgG1N297Q variant (BI-1607) with severely impaired Fc-binding to all FcγRs (‘αFcγRIIB_ΔFc_’) [8, 23]. At the time of generation, the αFcγRIIB_wt_ (BI-1206) antibody was intended to treat cancers in which FcγRIIB is expressed on tumor cells (i.e., B-cell cancers). In preclinical models, it demonstrated single-agent activity and enhanced the activity of tumor-directed antibodies such as αCD20 and αCD52 [8]. In contrast, the αFcγRIIB_ΔFc_ antibody (BI-1607) was designed to treat solid cancers, where FcγRIIB is expressed only on immune effector cells, and not tumor cells. Consistent with its highly specific blockade of FcγRIIB and impaired Fc:FcγR binding, this antibody lacked single-agent activity but enhanced the antitumor activity of αCD20, αHer2, and other tumor-targeting antibodies [8, 23].

To assess the therapeutic potential of FcγR blockade to enhance the activity of immune-modulatory antibodies, e.g., αPD-1 and αCTLA-4, in solid cancers, we generated surrogate blocking antibodies to mFcγRII, the murine homolog of hFcγRIIB and the sole inhibitory FcγR in the mouse [24]. These murine-specific reagents facilitate proof-of-concept studies in immune-competent syngeneic mouse tumor models. Fc:FcγR-proficient and -deficient blocking antibodies matching the human BI-1206 and BI-1607 clinical candidate antibodies to FcγRIIB were constructed by fusing Fv-sequences of the mouse FcγRII-specific antibody AT-130-2 to mouse IgG2a and mouse IgG1_N297A_ constant domains, respectively. All generated antibodies showed highly specific, high-affinity, Fv-mediated binding to mouse FcγRII, as assessed by surface plasmon resonance (SPR) with recombinant protein (Fig. S1A). Fc:FcγR proficiency and deficiency of the AT-130-2 mIgG2a and mIgG1_N297A_ variants (hereafter referred to as αFcγRII_wt_ and αFcγRII_ΔFc_, respectively) were similarly assessed by SPR using recombinant mouse activating and inhibitory FcγR proteins (Fig. S1A).

### αFcγRII_wt_ and αFcγRII_ΔFc_ differently modulate αPD-1 and αCTLA-4 antitumor activity in vivo

**Fig. 1:**
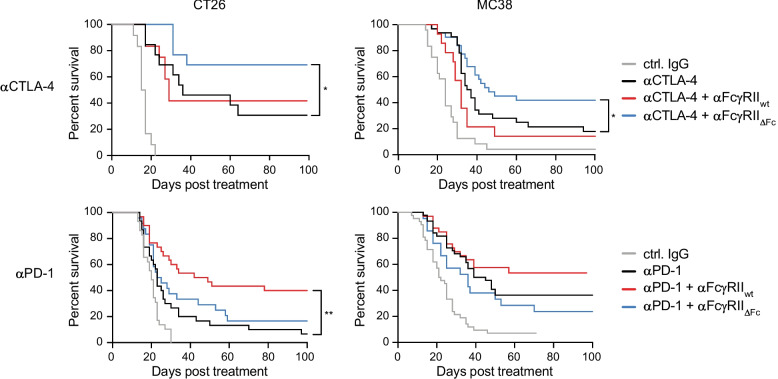
αFcγRII_wt_ and αFcγRII_ΔFc_ differently modulate αPD-1 and αCTLA-4-mediated antitumor activity. Survival in CT26 and MC38 tumor-bearing mice treated with αPD-1 or αCTLA-4 alone, or in combination with αFcγRII_wt_ or αFcγRII_ΔFc_, respectively. Treatment started when tumors had a volume of 50–100 mm^3 ^with n = 13–32 from 2–4 pooled experiments for αCTLA-4 in MC38, n = 12–13 mice from 2 pooled experiments for αCTLA-4 in CT26, n = 21–42 from 3–6 pooled experiments for αPD-1 in MC38, n = 29–30 from 5 pooled experiments for αPD-1 in CT26. *P < 0.05; **P < 0.01 by log-rank test

We first assessed whether αFcγRII_wt_ and αFcγRII_ΔFc_ could enhance the therapeutic activity of αCTLA-4 (clone 9H10) and αPD-1 (clone 29F.1A12) antibodies in immunocompetent mice bearing syngeneic CT26 colorectal tumors. This tumor model is infiltrated by immune cells, including CD8^+^ T cells, Tregs, and macrophages, and partially responds to αCTLA-4 but not, or poorly, to αPD-1 antibody therapy [25]. The model thus reflects the partial responsiveness to these agents observed in human cancer, leaving room for improvement in efficacy. Combination treatment with αFcγRII_ΔFc_, but not αFcγRII_wt_, significantly enhanced antitumor activity and survival in αCTLA-4-treated animals (Figs. 1 and S1B). Conversely, and of potential clinical significance, combined treatment with αPD-1 and αFcγRII_wt_, but not αFcγRII_ΔFc_, increased the proportion of mice cured in this otherwise αPD-1-resistant model (Figs. 1 and S1B).

Treatment of mice bearing MC38 colon adenocarcinoma tumors confirmed the inverse effects of αFcγRII antibody variants on αCTLA-4 and αPD-1 therapeutic activity (Fig. 1). Importantly, consistent with αFcγRII antibodies modulating the intrinsic activity of αCTLA-4 and αPD-1 and αCTLA-4 antibodies through FcγR blockade rather than direct anti-tumor activity, single-agent treatment with αFcγRII antibody variants did not affect tumor growth kinetics or survival (Fig. S1C).

### αFcγRII enhancement of αCTLA-4 requires an FcγR-silenced Fc-domain and correlates with Treg depletion and macrophage repolarization in vivo

**Fig. 2:**
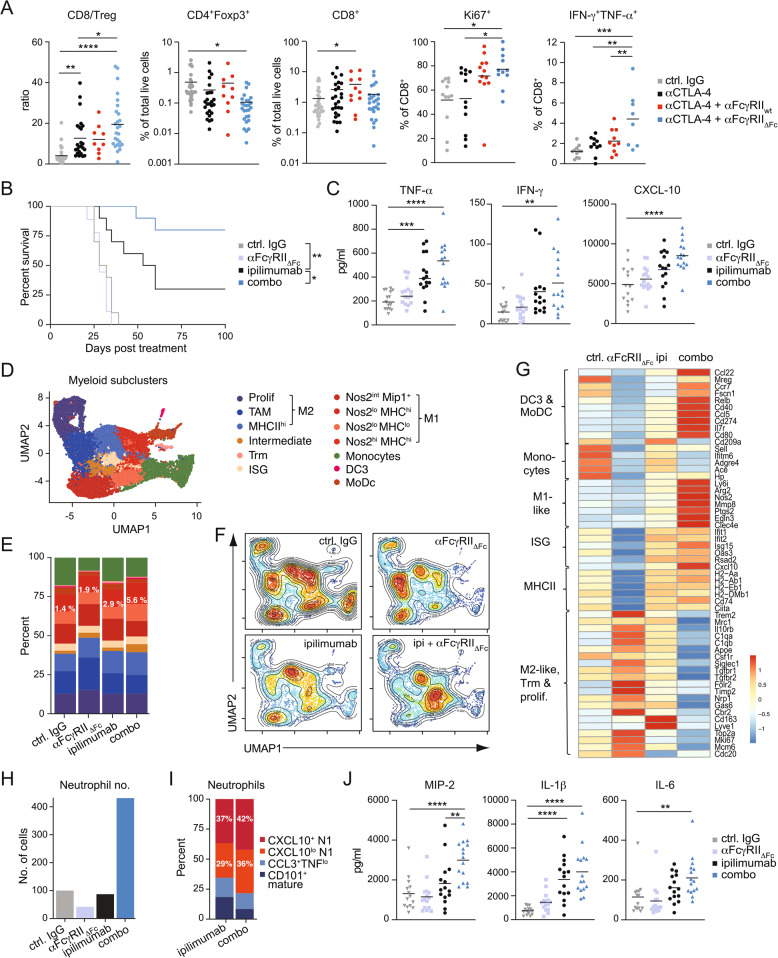
αFcγRII_ΔFc_ enhances αCTLA-4-induced TIL modulation. **A** Antibody-mediated TIL modulation in MC38 tumor-bearing C57BL/6 mice which received three treatments (with 10 mg/kg per antibody and injection) of αCTLA-4 alone or in combination with αFcγRII_wt_ or αFcγRII_ΔFc_ when tumors had reached ca. 150 mm^3^. Tumors were taken 24 h after the last treatment and assessed by flow cytometry. **B** Survival of MC38 tumor-bearing hCTLA4-transgenic C57BL/6 mice. When tumors were ca. 50 mm^3^, mice received 10 mg/kg of αFcγRII_ΔFc_, alone or in combination with 1 mg/kg of ipilimumab, compared to ipilimumab (1 mg/kg) alone or control IgG (10 mg/kg). **P < 0.01, *P < 0.05 by log-rank test. **C** Cytokine analysis in tumor from MC38 tumor-bearing hCTLA4-transgenic C57BL/6 mice treated as in B). Tumors were harvested 168 h after first treatment. Results are pooled from three different experiments with each symbol representing an individual mouse and lines representing mean. **** *P* < 0.0001, *** *P* < 0.001, **P < 0.01, *P < 0.05 by one-way ANOVA. **D**-**I** Single-cell RNA sequencing data from CD45^+^ sorted cells isolated from five MC38 tumors per group treated as in B). Tumors were harvested on day 7 after the first treatment. **D** Combined UMAP of myeloid subclusters color-coded by cell subsets. The identity of each cluster is based on DEGs shown in G) and Fig. S2G. **E** Proportions of each cluster across treatment groups, color-coded as in D) **F** Contour UMAP for each treatment group (**G**) Heatmap showing selected DEGs per treatment group (**H**) Bar-plots showing neutrophil numbers and **I** neutrophil subsets. **J** Cytokine analysis in tumor from MC38 tumor-bearing hCTLA4-transgenic C57BL/6 mice as in C)

Treg cells prevent excessive CD8^+^ T cell expansion in health [26], and Treg depletion enhances CD8^+^ T cell infiltration into tumors, promoting tumor rejection [13, 27–29]. Reported correlations between melanoma patient responses to the αCTLA-4 antibody ipilimumab and FcγR engagement, as well as the enhanced antitumor activity and Treg depletion of Fc-engaging humanized αCTLA-4 antibodies [11], prompted us to assess whether αFcγRII improved αCTLA-4 efficacy by promoting Treg depletion and improving intratumoral CD8:Treg ratios. In line with previous reports, treatment with αCTLA-4 alone improved CD8:Treg ratios, with trends toward reduced intratumoral Tregs and increased CD8^+^ T cell numbers (Figs. 2A and S2A). Consistent with FcγRII acting as a negative regulator of αCTLA-4-mediated Treg depletion and with Tregs suppressing CD8^+^ T cell function [30, 31], combined treatment with αFcγRII_ΔFc_ and αCTLA-4 mAb further improved CD8:Treg ratios and induced CD8^+^ T cell proliferation and activation (Fig. 2A).

Next, we extended our αFcγRII_ΔFc_-mediated αCTLA-4 mAb enhancement analyses to the clinical mAb ipilimumab. Human CTLA-4 transgenic (hCTLA-4) mice were transplanted with MC38 tumors and treated with ipilimumab, αFcγRII_ΔFc_, or a combination. Tumor growth and animal survival were assessed. Consistent with our murine αCTLA-4 surrogate antibody observations, αFcγRII_ΔFc_ enhanced ipilimumab in vivo efficacy, as shown by delayed tumor growth (Fig. S2B) and improved animal survival (*p* < 0.02, Log-rank Mantel-Cox test) (Fig. 2B). Animals cured by the αFcγRII_ΔFc_/ipilimumab combination rejected MC38 tumor cells upon rechallenge, demonstrating that immunological memory was induced (Fig. S2C). Consistent with our flow cytometry data from murine CTLA-4 mice, combined treatment with αFcγRII_ΔFc_ and ipilimumab activated effector T cells, as evidenced by induction of IFN-γ in tumors of hCTLA-4 transgenic mice with trends toward enhanced TNF-α and IFN-γ compared with αCTLA-4 single treatment (Figs. 2C and S2D). Moreover, consistent with enhancing effector CD8^+^ T cell responses, αFcγRII_ΔFc_ combined with ipilimumab induced the IFN-γ-responsive chemokine CXCL10 involved in recruiting activated T cells from lymph nodes to the tumor bed (Fig. 2C). We then performed single-cell RNA-seq to agnostically assess the mechanisms underlying the enhancement of αFcγRII_ΔFc_ in combination with ipilimumab. Unsupervised clustering and UMAP visualization of tumor-infiltrating leukocytes (TILs) harvested from antibody-treated MC38 tumor-bearing hCTLA-4 mice revealed the expected presence of multiple immune cell subsets, including B, T, NK cells, monocytes, macrophages, and neutrophils (Fig. S2E). Consistent with our observations in the murine CTLA-4/αCTLA-4 model system, αFcγRII_ΔFc_-enhanced ipilimumab efficacy correlated with more substantial Treg depletion, decreased exhausted CD8^+^ T cells, and increased activated CD8^+^ T cells (IFN-γ^+^ TNF-α^+^) in hCTLA-4 mice (Fig. S2F). Additionally, combined treatment with αFcγRII_ΔFc_ and ipilimumab increased activated Nos2^hi^ M1 macrophages (Figs. 2D- G and S2G) and induced DC3 and M1 macrophage gene signatures (Fig. 2G). In contrast, M2-related genes were downregulated (Fig. 2G). Combined αFcγRII_ΔFc_ and ipilimumab treatment increased the number of intratumoral ILC1 cells (Fig. S2H) and fourfold increased total neutrophils (PMN), with a preferential expansion of proinflammatory N1 PMNs, including those positive for CXCL10^+^, compared with the monotherapy group (Figs. 2H- I and S2I). Furthermore, consistent with FcγRII blockade enhancing ipilimumab through reprogramming myeloid cells, MIP-2, IL-1β, and IL-6 were induced or increased in tumors from animals treated with αFcγRII_ΔFc_ and ipilimumab, as quantified by multiplexed cytokine assay (Fig. 2J).

### αFcγRII_ΔFc_ enables low-dose αCTLA-4-based dual immune checkpoint blockade for rejection of poorly T cell-infiltrated tumors

**Fig. 3:**
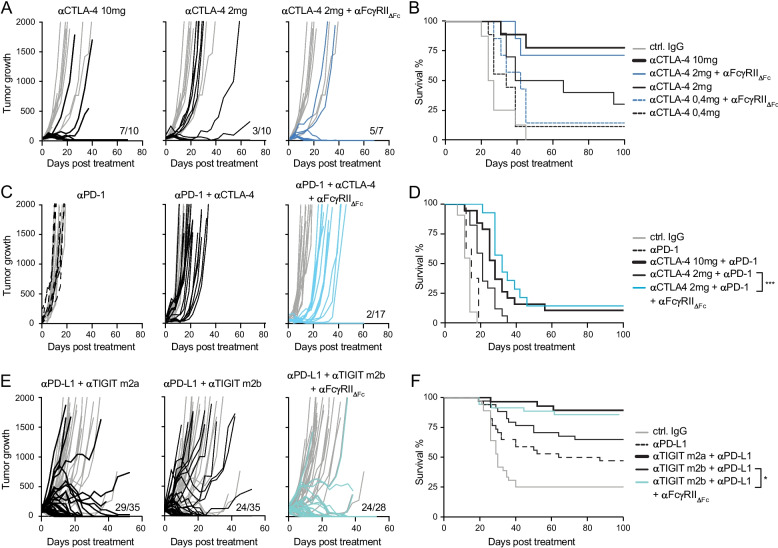
αFcγRII_ΔFc_ enhances both low-dose αCTLA-4 and αTIGIT treatment for tumor rejection. **A** Tumor growth and **B** survival of C57BL/6 mice bearing MC38 tumors. Mice were treated three times with indicated doses of αCTLA-4, alone or in combination with αFcγRII_ΔFc_ with the control IgG also shown. Tumors were ca. 50 mm^3^ at start of treatment. Numbers in the graph indicate totally cured mice/total number of mice in the group for the αCTLA4 treatments. One representative experiment out of 3 is shown. **C** Tumor growth and **D** survival of C57BL/6 mice bearing B16 tumors. Mice were treated when tumors were ca. 50 mm^3^ with 2 or 10 mg/kg of αCTLA-4 and αPD-1 (10 mg/kg), combined with αFcγRII_ΔFc_ when indicated. Results are pooled from 2 experiments with n = 17–20 mice per group. ***P < 0.001 using log-rank test. **E** Tumor growth and **F** survival of C57BL/6 mice bearing CT26 tumors. Mice were treated three times when tumors were ca. 100–150 mm^3^ with 10 mg/kg of αTIGIT and αPD-L1 (10 mg/kg), combined with 20 mg/kg of αFcγRII_ΔFc_ when indicated. Results are pooled from 4 experiments with n = 28–35 mice per group. *P < 0.05 using log-rank test

Dual treatment with αCTLA-4 and αPD-1 mAb increases ICB efficacy [32]. It is required for activity in particularly resistant cancer patients, e.g., those with mismatch-repair proficient colorectal cancer [33] and those who have relapsed on prior ICB therapy [3]. However, dose-dependent toxicity limits the use of available αCTLA-4 mAb-based regimens [34–36]. Given αFcγRII_ΔFc_’s enhancing effects on αCTLA-4 efficacy and Treg depletion, we tested whether it could increase the efficacy of safe, low doses of αCTLA-4. αCTLA-4 dose-dependent antitumor activity was confirmed by treating MC38 tumor-bearing mice with 10, 2, or 0.4 mg/kg of antibody (Fig. 3A and B). As expected, maximal efficacy was observed with 10 mg/kg αCTLA-4, equivalent to the highest tested, maximally efficacious, but toxic dose in human cancer patients. Dosing at 2 mg/kg, intermediate between the two clinically approved αCTLA-4 doses of 1 and 3 mg/kg, was also efficacious, whereas 0.4 mg/kg had little or no antitumor activity. Strikingly, coadministration of αFcγRII_ΔFc_ with the clinically relevant 2 mg/kg dose of αCTLA-4 enhanced efficacy to that of the maximally efficacious but toxic 10 mg/kg dose.

We next assessed whether adding αFcγRII_ΔFc_ to a combination of low-dose αCTLA-4 mAb and maximally efficacious αPD-1 mAb could enable rejection of poorly CD8^+^ T cell-infiltrated B16 tumors transplanted into syngeneic C57BL/6 mice. The highly resistant nature of this cancer model was confirmed by the absence of surviving mice after treatment with a control antibody, low-dose αCTLA-4, αPD-1, or the combination of low-dose αCTLA-4 and αPD-1. Again, adding αFcγRII_ΔFc_ to low-dose αCTLA-4/αPD-1 converted the regimen into an effective one, as shown by delayed tumor growth (Fig. 3C) and an increased proportion of surviving mice (Fig. 3D).

### Selective FcγRII blockade enhances αTIGIT

Like αCTLA-4, αTIGIT antibodies were recently shown to confer FcγR-dependent antitumor activity, thereby improving the efficacy of αPD-L1 immune checkpoint blockade [17]. αTIGIT efficacy and tumor microenvironment remodeling were greater with high A:I FcγR engagement ratio antibody isotypes, suggesting that activating FcγRs augment antitumor activity and that FcγRIIB reduces it. We therefore tested whether selective FcγRIIB blockade with αFcγRII_ΔFc_ could enhance αTIGIT during αPD-L1-based immune checkpoint blockade, using an analogous experimental setup [17]. Tumor-bearing CT26 mice were treated with αTIGIT and αPD-L1, with or without αFcγRII_ΔFc_. For the TIGIT antibody, we chose the mIgG2b isotype to mimic the balanced FcγR A:I engagement ratio of clinically relevant FcγR-engaging αTIGIT antibodies, e.g., tiragolumab. For the αPD-L1 antibody, the weakly FcγR-engaging mIgG1 isotype was used. As observed with αCTLA-4, FcγRII blockade with αFcγRII_ΔFc_ enhanced antitumor activity and improved survival in αTIGIT + αPD-L1-treated animals (Fig. 3E and F). Consistent with a high A:I FcγR engagement ratio determining αTIGIT efficacy, combined treatment with αTIGIT mIgG2b antibody and FcγRII blockade similarly enhanced αPD-L1 compared to a mIgG2a αTIGIT antibody with a high FcγR A:I engagement ratio (Fig. 3E and F).

### αFcγRII enhancement of αPD-1 mAb requires an FcγR-binding Fc-domain

αPD-1/PD-L1 antibodies, owing to their broad efficacy and favorable tolerability, constitute the backbone of ICB and are approved for more than 40 cancer types [1]. Most approved αPD-(L)1 regimens do not include αCTLA-4 or other immune checkpoint inhibitors. Consequently, drugs that enhance αPD-1 and overcome αPD-1 resistance address a significant unmet medical need. Our data indicated that αFcγRII_ΔFc,_ while potently enhancing αCTLA-4 monotherapy and CTLA-4/PD-1 combination ICB, did not modulate or enhance αPD-1 (Fig. 1). Instead, only the αFcγRII_wt_ antibody variant, equipped with an FcγR-binding Fc domain, improved αPD-1 efficacy. These observations prompted us to analyze the mechanisms underlying αFcγRII_wt_’s αPD-1-enhancing effects.

**Fig. 4:**
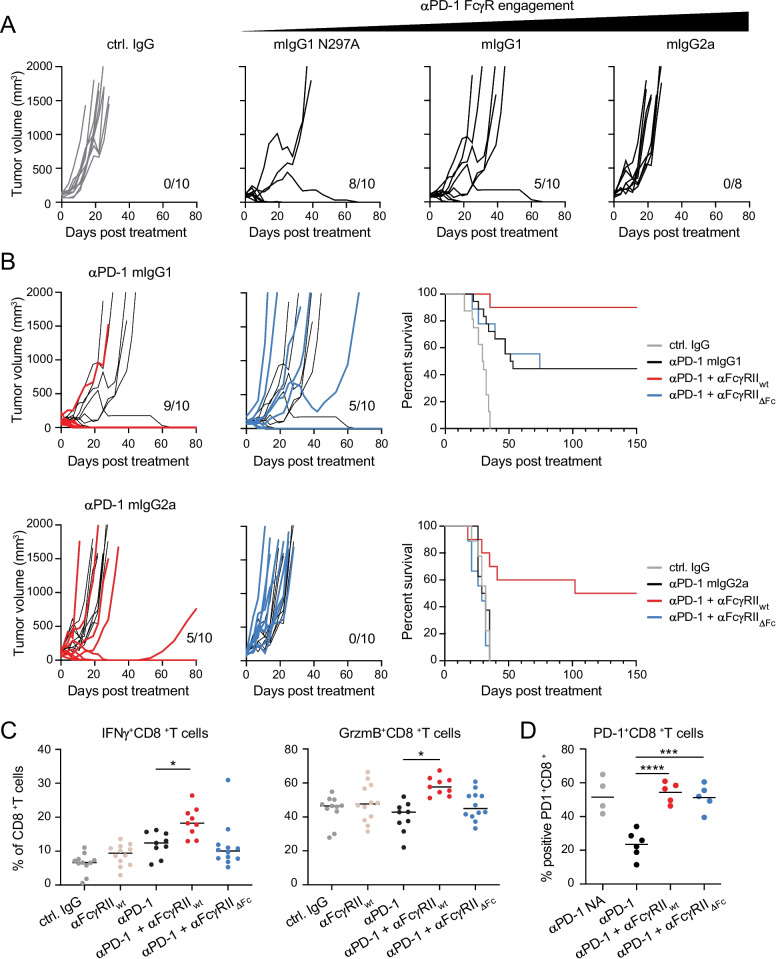
αFcγRII_wt_ enhances αPD-1 treatment for tumor rejection. **A** Tumor growth in MC38 tumor-bearing mice treated three times with αPD-1 (10 mg/kg) in different isotype formats compared to negative control IgG, alone or **B** in combination with 10 mg/kg of αFcγRII_wt_ or αFcγRII_ΔFc_. Tumors were ca. 100 mm^3^ at start of treatment. Numbers in the graph indicate totally cured mice/total number of mice in the group. **C** MC38 tumor-bearing mice were treated with 25 mg/kg of αFcγRII_ΔFc_ or αFcγRII_wt_ in combination with αPD-1 in comparison to αPD-1, αFcγRII_wt_ or control IgG alone on day 0, 3 and 7. Tumors were analyzed by flow cytometry on day 4 and 8 after treatment start. Each dot represents one mouse. **P < 0.01, *P < 0.05 using ordinary one-way ANOVA. **D** In vivo trogocytosis of αPD-1 from CD8^+^ T cells. MC38 tumor-bearing mice were treated (i.v) with αPD-1 or αPD-1-N297A labeled with AF647. Two groups were pretreated with αFcγRII_ΔFc_ or αFcγRII_wt_. The CD8^+^ cells in the tumors were analyzed for αPD-1 staining 1 h post injection. Each dot represents one mouse. ****P < 0.0001, ***P < 0.001 by ordinary one-way ANOVA

We first confirmed previous observations that FcγR-engagement reduces αPD-1 efficacy, using αPD-1 variant antibodies that preferentially engage activating FcγRs (mIgG2a, high FcγR A: I engagement ratio), the inhibitory FcγRII (mIgG1, low FcγR A: I engagement ratio), or no FcγRs (mIgG1_N297A_). Accordingly, in vivo αPD-1 efficacy ranked mIgG1_N297A_ > mIgG1 > > mIgG2a, i.e., in order of decreasing FcγR engagement, as demonstrated by reduced tumor growth and improved survival in MC38 tumor-bearing animals (Fig. 4A). Next, we assessed the protective effects of αFcγRII_wt_ and αFcγRII_ΔFc_ on αPD-1 in vivo antitumor activity using weakly (mIgG1) or strongly (mIgG2a) activating FcγR-engaging αPD-1 variants. As observed with the hIgG_4_-mimicking, moderately FcγR-engaging, rIgG2a αPD-1 antibody, combined treatment with αFcγRII_wt_, but not αFcγRII_ΔFc_, enhanced the efficacy of the weakly FcγR-engaging αPD-1 mIgG1 and, remarkably, overcame resistance to the strongly FcγR-engaging αPD-1 mIgG2a antibody, as assessed by reduced tumor growth and improved animal survival (Fig. 4B).

Our finding that αFcγRII antibodies required an FcγR binding Fc domain to enhance αPD-1 demonstrated that αFcγRII-blockade alone was insufficient to enhance αPD-1. Previous reports indicated roles for both activating and inhibitory FcγRs, limiting αPD-1 therapeutic efficacy through macrophage-mediated depletion (ADCP) of αPD-1-coated CD8^+^ T cells and trogocytosis (transfer) of T cell-bound αPD-1 antibodies from CD8^+^ T cells, respectively. We assessed the relevance of these mechanisms to αFcγRII modulation of αPD-1 in vivo*.* Combined treatment with αFcγRII_wt_, but not αFcγRII_ΔFc_, and αPD-1 increased intratumoral activated CD8^+^ T cells (Figs. 4C and S3A). In contrast, both αFcγRII_wt_ and αFcγRII_ΔFc_ similarly prevented in vivo macrophage-mediated trogocytosis of αPD-1 from CD8^+^ T cells (Fig. 4D).

### Human αFcγRIIB_wt_ blocks macrophage phagocytosis of nivolumab and pembrolizumab-treated T cells with clinically relevant PD-1 expression

**Fig. 5:**
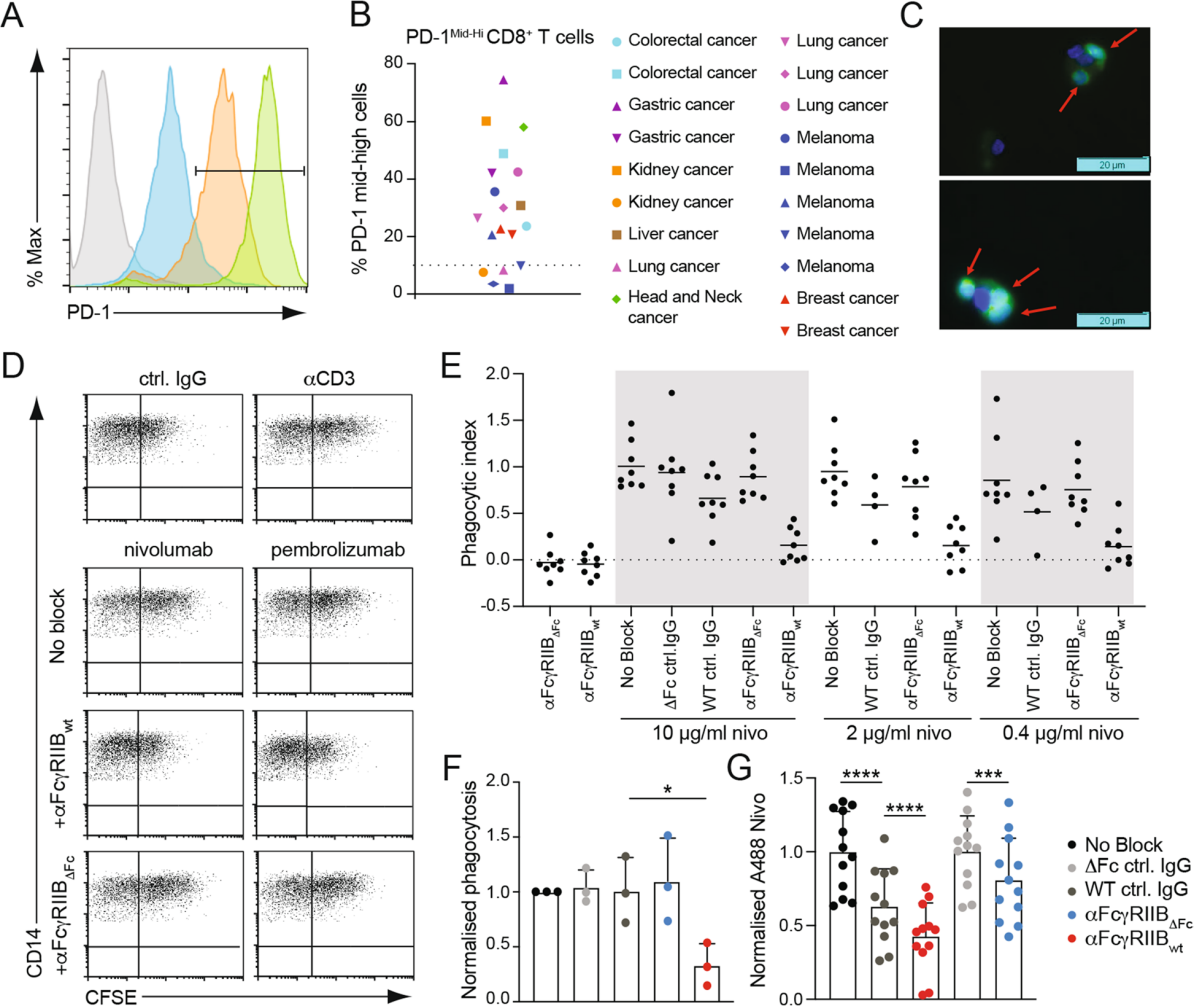
Human αFcγRIIB_wt_ blocks phagocytosis of αPD-1 treated T cells. **A** Transfected Jurkat cells expressing different levels of PD-1. Blue = low; orange = mid; high = green. **B** % intratumoral CD8^+^ T cells in different human tumor tissues expressing PD-1 and equivalent levels to mid-high Jurkat cells. **C** Fluorescence microscopy pictures of αPD1-mediated phagocytosis. PD-1 expressing Jurkat cells (CFSE-labelled; green) were opsonized with nivolumab and co-cultured with FcγR-expressing monocyte-derived macrophages (MDM). CFSE^+^CD14^+^ cells were FACS sorted onto microscope slides and counterstained with DAPI. Arrows indicate phagocytosed target cells. **D**-**E** CFSE-labelled, αPD-1 opsonized Jurkat-hPD1 high cells were incubated with αFcγRIIB-treated or untreated MDMs and **D** assessed by flow cytometry for the level of phagocytosis, determined as CFSE^+^ CD14^+^ macrophages as a percentage of total macrophages. **E** Results presented for titrated doses of nivolumab, normalized to control (αCD3 hIgG1 = 1) with each point representing an MDM donor. **F** Assay was performed as in D-E) using in vivo αCD3/αCD28 dynabeads activated human T cells and donor matched MDM. Results are shown as degree of target cell phagocytosis normalized to control. Each dot represents one T cell donor. **G** αPD-1 trogocytosis. Jurkat cells expressing human PD-1 were opsonised with A488-labelled nivolumab and co-cultured with MDM. Trogocytosis was assessed as the level of A488-labelled αPD-1 on macrophages determined by flow cytometry. Each point represents a single MDM donor. ****P < 0.0001, ***P < 0.001*P < 0.05 by one-way ANOVA

Our results demonstrated that αFcγRIIB antibodies required an FcγR-binding Fc domain to enhance αPD-1 in vivo and suggested that αFcγRIIB_wt_ achieves this by sparing αPD-1-coated CD8^+^ T cells from macrophage-mediated depletion rather than by reducing the transfer (trogocytosis) of αPD-1 antibodies from T cells to macrophages. We assessed the human relevance of these mechanisms for αFcγRIIB modulation of αPD-1 using a co-culture system in which Jurkat cells expressing intratumorally relevant levels of PD-1 (Fig. 5A and B) were co-cultured with FcγR-expressing monocyte-derived macrophages (MDM) (Fig. 5C). Jurkat-PD-1/macrophage co-cultures were treated with human αPD-1 antibodies nivolumab or pembrolizumab in the presence or absence of clinically relevant human αFcγRIIB_wt_ (BI-1206) or αFcγRIIB_ΔFc_ (BI-1607) mAb, and macrophage phagocytosis of Jurkat-PD-1 cells (Fig. 5D-F) or macrophage trogocytosis of αPD-1 from Jurkat-PD-1^+^ T cells (Fig. 5G) was assessed. The human αPD-1 antibodies mediated macrophage phagocytosis of Jurkat-PD-1 cells. Strikingly, αFcγRIIB_wt_ (BI-1206) but not αFcγRIIB_ΔFc_ (BI-1607) completely blocked αPD-1-mediated phagocytosis (Fig. 5D-E). Moreover, a similar result was observed using in vitro-activated primary human T cells as targets, where only αFcγRIIB_wt_ inhibited αPD-1-mediated phagocytosis of target cells (Fig. 5F). In contrast, and as observed in vivo, both αFcγRIIB_wt_ (BI-1206) and αFcγRIIB_ΔFc_ (BI-1607) reduced αPD-1 trogocytosis, as shown by decreased αPD-1 mAb transfer to the macrophage surface (Fig. 5G).

### Immune checkpoint subcellular expression determines FcγR blockade requirements

Our results demonstrated that tailored FcγR blockade effectively enhances the in vivo therapeutic activity of immune checkpoint-blocking antibodies. We and others have previously shown that intratumoral Tregs express higher levels of CTLA-4 than CD8^+^ T cells [11, 12, 37]. Accordingly, Tregs are more sensitive to depletion by an αCTLA-4 antibody. Conversely, intratumoral CD8^+^ T cells express higher levels of PD-1 than Tregs and are therefore more sensitive to depletion by an αPD-1 antibody [12] (Figs. 4C and S3B-C). Accordingly, Fc:FcγR-silenced αFcγRII enhanced αCTLA-4, whereas Fc:FcγR-proficient αFcγRII boosted αPD-1, correlating with enhanced and blunted depletion of CTLA-4^High^ Treg cells and PD-1^High^ effector CD8^+^ T cells [11, 12, 37], respectively. Neither FcγRII-blocking antibody variant showed single-agent antitumor activity or modulated TIL numbers, suggesting that the observed effects did not result from depletion of FcγRII-expressing cells themselves but rather from modulation of αCTLA-4 and αPD-1 antibody interactions with FcγRs. We therefore hypothesized that the differential boosting effects of FcγR-silenced and FcγR-proficient αFcγRII in each case related to their ability to either bind and block FcγRII selectively through Fv-mediated interactions (Fc-silenced αFcγRII) or bind and block all FcγR through a combination of Fv- and Fc-mediated interactions (Fc:FcγR-proficient αFcγRII) once bound to the target cell surface through FcγRII.

**Fig. 6:**
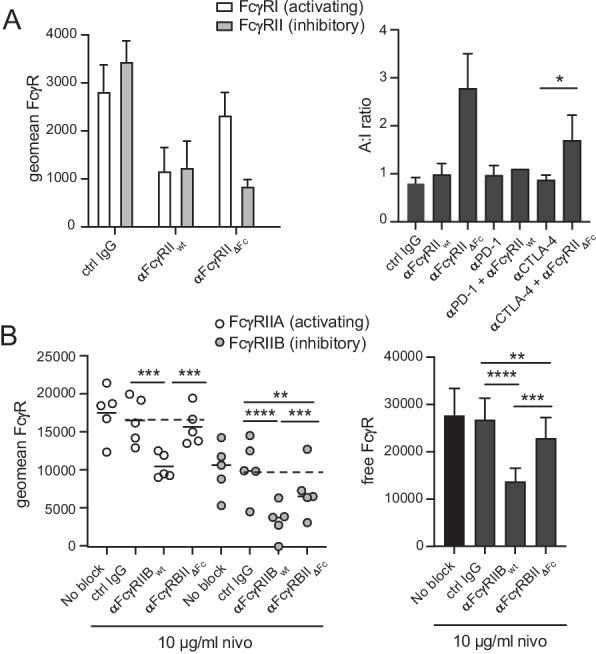
Access or blockade of FcγR in vitro and in vivo determines ICB outcomes. **A** MC38 tumor-bearing C57BL/6 mice received three treatments (with 10 mg/kg per antibody per injection) when tumors reached ca. 150 mm^3^. Tumors were taken 24 h after the last treatment, and FcγRs on myeloid cells assessed by flow cytometry. Free, unbound, activating FcγRs and inhibitory FcγRII, and the resulting A:I ratio following treatment with αFcγRII_wt_ or αFcγRII_ΔFc_. *P < 0.05 by Student’s t-test. **B** CFSE-labelled, αPD-1 opsonized Jurkat-hPD1 high cells were incubated with αFcγRIIB-treated or untreated MDM. Following co-culture, MDM were stained with αFcγRIIB and αFcγRIIA antibodies and analysed by flow cytometry. Geometric mean of individual receptors is shown in the left graph and total fluorescence in the graph to the right ****P < 0.0001, ***P < 0.001, **P < 0.01 by 2way ANOVA

We tested this in vivo and in vitro, assessing the availability of free, unbound, activating Fc gamma receptors and FcγRIIB, and the resulting A:I ratio on macrophages following treatment with αFcγRII(B)_wt_ or αFcγRII(B)_ΔFc_ mAb. Consistent with our hypothesis, macrophages isolated from animals treated with αFcγRII_ΔFc_ showed improved A:I ratios, whereas macrophages treated with FcγRII_wt_ showed blunted FcγR availability (Fig. 6A). The analogous findings were made with the human-relevant therapeutic antibodies in the T cell/macrophage co-culture system in vitro. While the Fc:FcγR-impaired αFcγRIIB antibody BI-1607 improved A:I ratios by selectively blocking the inhibitory FcγRIIB, the Fc:FcγR-proficient αFcγRIIB antibody BI-1206 reduced both inhibitory and activating macrophage FcγRs (Fig. 6B).

## Discussion

Several activating and a single inhibitory FcγR regulate the efficacy of tumor-targeting and immune-modulatory antibodies by promoting and reducing, respectively, Fc-dependent activation of FcγR-expressing immune effector cells [9, 10]. Accordingly, preclinical and clinical data demonstrate that αCTLA-4, αTIGIT, and other antibodies depend on activating FcγRs to trigger Treg depletion [13], myeloid reprogramming, and robust CD8^+^ T cell antitumor immunity [14], mechanisms that underlie improved survival in ICB-responding cancers such as lung cancer [17]. Critically, FcγR-dependent mechanisms may extend the clinical benefit of ICB to otherwise resistant cancers, e.g., microsatellite stable colorectal cancer [33], and to patients who have relapsed on αPD-1 [3]. Accordingly, Fc-engineered FcγR-enhanced αCTLA-4 mAbs are being developed to treat poorly immunogenic and ICB-refractory cancers [38].

We are exploring tailored FcγR blockade as an alternative to Fc engineering to improve FcγR engagement. Here, we showed that FcγR blockade with Fc-mutant and wild-type αFcγRIIB antibodies enhanced αCTLA-4 and αPD-1 efficacy, respectively. The ability of αFcγRIIB_ΔFc_ to enhance αCTLA-4 mAb efficacy matched αCTLA-4’s known reliance on activating FcγRs, correlating with deeper Treg depletion, myeloid reprogramming, and CD8^+^ T cell activation. Interestingly, αFcγRIIB_ΔFc_ also induced intratumoral CXCL10, part of the CXCL9/10/11-CXCR3 axis responsible for recruiting CD8^+^ and CD4^+^ T cells from lymph nodes to the tumor bed [39], predicting response to ICB [40], and extending ICB activity to otherwise resistant, poorly CD8^+^ T cell-infiltrated “cold” tumors [41]. Accordingly, αFcγRIIB_ΔFc,_ besides enhancing low-dose αCTLA-4 efficacy to fully therapeutic levels in the ICB partially responsive MC38 tumor model, extended its activity to the poorly CD8^+^ T cell-infiltrated ICB-resistant B16 tumor model. Without FcγRIIB blockade, such efficacy required an αCTLA-4 dose of 10 mg/kg, which is a clinically prohibitive dose owing to its severe toxicity [42]. Whether CTLA-4 mAb efficacy can be separated from its toxicity remains a topic of debate [43–45]. While only human clinical trials can answer this question, given the lack of tox-predictive mouse models, independent observations support the clinical relevance of low-dose αFcγRIIB_ΔFc_-enhanced αCTLA-4. Notably, hIgG1 ipilimumab and hIgG2 tremelimumab show similar toxicities at comparable exposure levels [34–36], suggesting that systemic blockade of the CTLA-4:B7 immune checkpoint, rather than FcγR engagement, is the primary cause of toxicity.

In contrast, as discussed above, preclinical and clinical data demonstrate that tumor-associated FcγR-engagement enhances αCTLA-4 efficacy and is required for activity in poorly CD8^+^ T cell-infiltrated and ICB-refractory cancers [11, 37]. Notably, FcγRIIB is upregulated in the tumor microenvironment but shows limited expression on blood myeloid cells and is not expressed on NK cell effectors [8, 11, 46, 47]. Relative to Fc-enhanced αCTLA-4 mAbs, which are engineered to bind strongly to the activating FcγRIIA and FcγRIIIA ubiquitously expressed in blood, targeting FcγRIIB may offer a more tumor-selective approach to enhance antibody immunotherapy. We recently demonstrated the safety of this approach in the context of tumor-targeting antibody immunotherapy by co-administering the FcγRIIB-blocking antibody BI-1607 with trastuzumab in breast cancer patients [23].

While combined ICB with FcγR-engaging mAb, e.g., αCTLA-4, is required for efficacy in poorly CD8^+^ T cell-infiltrated tumors, αPD-(L)1 single ICB, owing to its superior tolerability, is the most widely approved ICB and the preferred treatment in T cell-inflamed cancers. Enhancing efficacy and overcoming resistance to αPD-1 ICB has tremendous medical potential. In this setting, we find that αFcγRIIB_wt_ enhances therapeutic efficacy by blunting αPD-1 engagement of both inhibitory and activating FcγRs. This was demonstrated by αFcγRIIB_wt_ exclusively restoring and enhancing FcγR-engaging αPD-1 antibodies in vivo and by sparing activated PD-1 high-expressing T cells treated with pembrolizumab or nivolumab from macrophage phagocytosis in vitro and in vivo. These observations were consistent with the differential effects of αFcγRII_ΔFc_ and αFcγRII_wt_ on αCTLA-4, with αFcγRII_ΔFc_ promoting, and αFcγRII_wt_ reducing αCTLA-4 antibody-mediated ADCP, reflected by decreased Treg and increased CD8^+^ T cells following combination therapy in vivo. In contrast, both antibodies reduced macrophage-mediated αPD-1 antibody trogocytosis. Together, these observations suggest that αFcγRII_wt_ primarily enhances αPD-1 efficacy by reducing macrophage-mediated ADCP of PD-1^+^ CD8^+^ T cells, rather than by preventing αPD-1 trogocytosis. This notion is consistent with earlier observations that both activating and inhibitory FcγRs mediate trogocytosis [48], and align with previous observations that macrophage-dependent phagocytosis controls antibody efficacy and target cell depletion in vivo [12, 49–54]. A detrimental role for FcγRs in αPD-1-based therapy has been previously suggested, including with human-relevant αPD-1 antibodies of the hIgG4 isotype [18, 19, 55]. Consistent with the hIgG4 isotype being a relatively poor engager of FcγRs compared with hIgG1, we find that macrophage phagocytosis of human T cells coated with nivolumab or pembrolizumab required robust yet clinically relevant PD-1 expression. In contrast to CTLA-4, which is most highly expressed on intratumoral Treg cells, PD-1 is progressively increased with activation on effector CD8^+^ T cells and in exhausted CD8^+^ T cell states sensitive to αPD-1 ICB. Sparing and maximizing the activity of these critical effector cells, e.g., by minimizing FcγR-dependent depletion, is highly warranted. Interestingly, while intratumoral anti-PD-1 engagement is associated with deleterious effects that compromise efficacy, recent data suggest that low anti-PD-1 engagement of FcγRs in tumor-draining lymph nodes (TDLN) may be beneficial [56]. FcγR engagement was shown to mobilize immune cells, including tumor-specific CD8 + T cells, in the TDLN. Increased CD8 + T cell mobilization was associated with improved therapeutic responses. Our tailored approach to FcγR blockade provides an attractive strategy for spatiotemporally tuning FcγR engagement. It would be interesting to assess whether staggered treatment with a single-agent Fc-engaging αPD-1, followed by combined therapy with FcγRIIBwt, could enhance antitumor CD8 + T cell expansion and αPD-1 efficacy.

Taken together, our results show that FcγRIIB-selective blockade promotes ADCP, whereas pan-FcγR blockade prevents it. Accordingly, our findings suggest that the differential abilities of the antibodies to enhance anti-CTLA-4 and anti-PD-1 efficacy are due to CTLA-4 and PD-1 being more highly expressed on Tregs and CD8^+^ T cells, and Tregs and CD8^+^ T cells being sensitive to αCTLA-4- and αPD-1-mediated ADCP, respectively [11, 12, 37].

## Conclusions

In conclusion, we provide preclinical proof of concept that tailored FcγRIIB blockade with Fc-mutated or wild-type antibodies achieves highly specific FcγRIIB or pan-FcγR blockade, thereby enhancing the efficacy of clinically relevant αCTLA-4 and αPD-1 ICB through distinct mechanisms that respectively enhance or block antibody-mediated ADCP of Treg and CD8^+^ T cells.

Human Fc-tailored αFcγRIIB antibodies BI-1607 (NCT06784648) and BI-1206 (NCT04219254, NCT03571568), which have the potential to enhance ICB efficacy and extend its use to currently untreatable, resistant cancers, are in clinical development.

## Supplementary Information

Additional file 1. Supplemental figures and tables.

## Data Availability

The processed data and raw data (single cell RNA sequencing) have been uploaded to the NCBI Gene Expression Omnibus (GEO) database (https://www.ncbi.nlm.nih.gov/geo/) with accession number GSE335831.

## Abbreviations

FcγR: Fc gamma receptor
ICB: Immune checkpoint blockade
mAb: Monoclonal antibody
