# Supplementary material for: Tailored FcγR blockade enhances immune checkpoint therapy and overcomes resistance
Source: J Exp Clin Cancer Res. 2026 Jul 25;45:167. doi: 10.1186/s13046-026-03785-5 (PMC13418441; doi:10.1186/s13046-026-03785-5)
Supplement: Supplementary file 1 — Additional file 1. Supplemental figures and tables. [file 13046_2026_3785_MOESM1_ESM.docx]

**Supplementary Materials**

**Table S1. Flow cytometry antibodies**

| **Marker** | **Species** | **Fluorophore(s)** | **Clone** | **Supplier** |
| --- | --- | --- | --- | --- |
| CD14 | Human | APC/BV421 | M5E2 | Biolegend |
| PD-1 | Human | PE | EH12.2H7 | Biolegend |
| PD-1 | Human | Alexa647 | 1D6E10 | In-house (BioInvent) |
| CD45 | Human | Alexa700 | HI30 | BD Biosciences |
| CD3 | Human | PercP-Cy5.5 | OKT3 | BD Biosciences |
| CD8 | Human | BV605 | SK1 | BD Biosciences |
| PD-1 | Mouse | BV421 | RMP1-30 | Biolegend |
| CD62L | Mouse | Suberbright600 | MEL-14 | ThermoFisher |
| CD3 | Mouse | BV750  APC-Fire750 | 17A2  KT3.1.1 | Biolegend |
| FoxP3 | Mouse | Alexa488; PE | FKJ-16s | ThermoFisher |
| CD45.2 | Mouse | BB700 | 104 | BD Biosciences |
| CD4 | Mouse | PE/Texas Red  BV750 | RM4-5  GK1.5 | ThermoFisher  Biolegend |
| Ki67 | Mouse | Alexa647 | B56 | BD Biosciences |
| CD8 | Mouse | Alexa700 | 53-6.7 | Biolegend |
| IFN−γ | Mouse | BV421 | XMG1.2 | BD Biosciences |
| CD25 | Mouse | BV605 | PC61 | Biolegend |
| Granzyme B | Mouse | Alexa488 | QA18A28 | Biolegend |
| CX3CR1 | Mouse | PE-Fire810 | SA011A11 | Biolegend |
| TNF | Mouse | APC | MP6-XT22 | BD Biosciences |
| FcγRI | Human | Alexa647 | AT166-2 (F(ab’)_2_) | In-house (Southampton) |
| FcγRIIa | Human | Alexa647 | E08 (hIgG1 N297Q) | In-house (Bioinvent) |
| FcγRIIb | Human | Alexa647 | 6G11 (hIgG1 N297Q) | In-house (Bioinvent) |
| FcγRIII | Human | Alexa647 | 3G8 (F(ab’)_2_) | In-house (Southampton) |


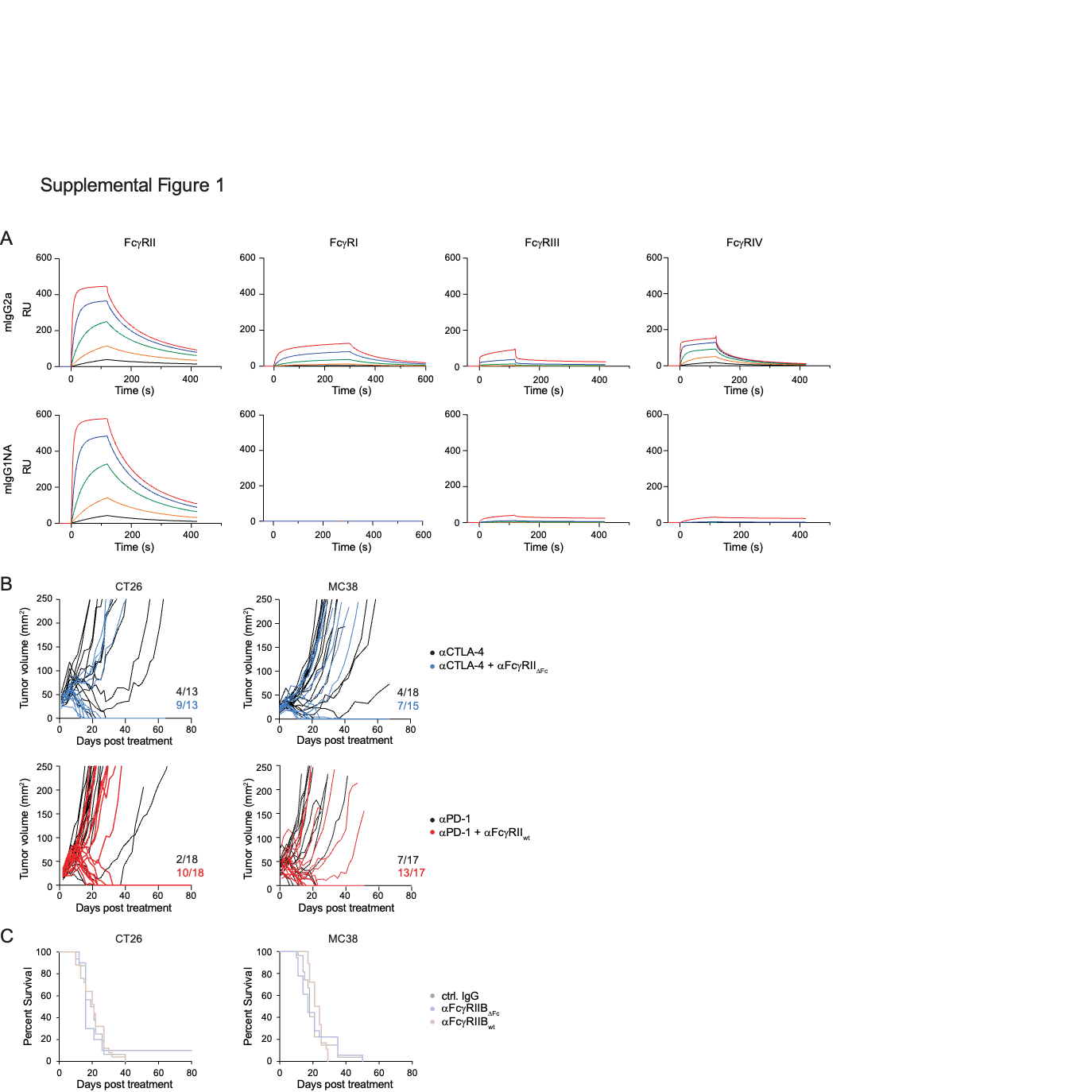


**Fig. S1** αFcγRII_ΔFc_ (mIgG1NA) or αFcγRII_wt_ (mIgG2a) specifically bind to FcγRII and induce antitumor activity in combination with αCTLA-4 and αPD-1, respectively. **(A)**Biacore analysis of immobilised αFcγRII_ΔFc_ or αFcγRII_wt_ binding to mouse FcγR proteins demonstrating antibodies’ specificity. 5-fold dilutions of FcγR were used starting at 100nM for FcγRI and 1000nM for all other FcγR. Representative of 2 independent experiments.  **(B)** Tumor growth curves underlying survival curves shown in Fig. 1. Numbers in the graph indicate totally cured mice / total number of mice in the group. **(C)** Single-agent activity of αFcγRII_ΔFc_ and αFcγRII_wt_ in CT26 and MC38, related to Fig. 1.

**
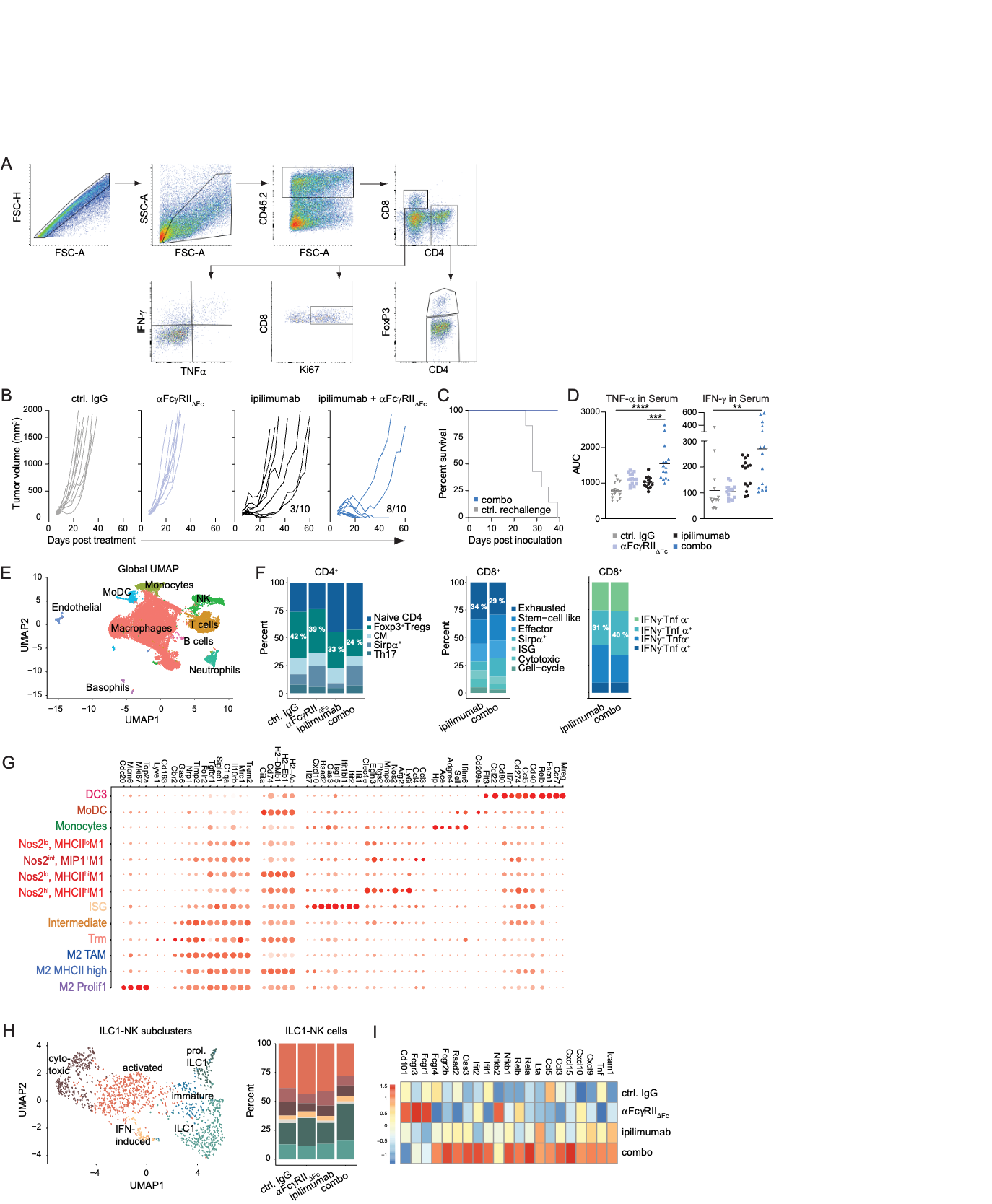
**

**Fig. S2** αFcγRII_ΔFc_ enhances ipilimumab-induced antitumor activity in MC38 tumor-bearing hCTLA-4 transgenic mice. Related to Fig. 2. **(A)**Flow cytometry gating strategy to assess antibody-mediated TIL modulation in MC38 tumor-bearing C57BL/6 mice as shown in Fig. 2A.**(B)**Tumor growth curves underlying survival graphs in Fig. 2B. Numbers in the graph indicate totally cured mice / total number of mice in the group.**(C)** Survival following tumor rechallenge in previously surviving mice from Fig. 2B. Control mice had not previously been challenged or treated. **(D)** Cytokine analysis in serum from MC38 tumor-bearing hCTLA4-transgenic C57BL/6 mice treated as in Fig. 2B. Serum was collected 24, 72, 168 h after treatment start. The increase in IFN-γ and TNF-α in the serum was quantified for each mouse by measuring the area under the curve during the time of the treatment. Results are pooled from three different experiments with each symbol representing an individual mouse and lines representing mean. **** P < 0.0001, *** P < 0.001, **P < 0.01by one-way ANOVA. **(E-I)** scRNA-seq data from CD45^+^ sorted cells isolated from MC38 tumors, related to Fig. 2D-I. **(E)** The UMAP clustering of 26065 cells coloured by immune cell clusters originating from 4 treatments (ctrl. IgG, αFcγRII_ΔFc_, ipilimumab, combination of αFcγRII_ΔFc_ and ipilimumab). Each treatment group contain 5 mice/group. **(F)** CD4^+^(left panel) and CD8^+^ (right panel) T cell distribution **(G)** marker gene expression across myeloid cells. **(H)**UMAP clustering of NK and ILC1 cells by subset, with their quantification across treatment groups shown in the bar plot. **(I)** Heatmap on neutrophils showing the relative expression of genes associated with protumorigenic or antitumorigenic functions.


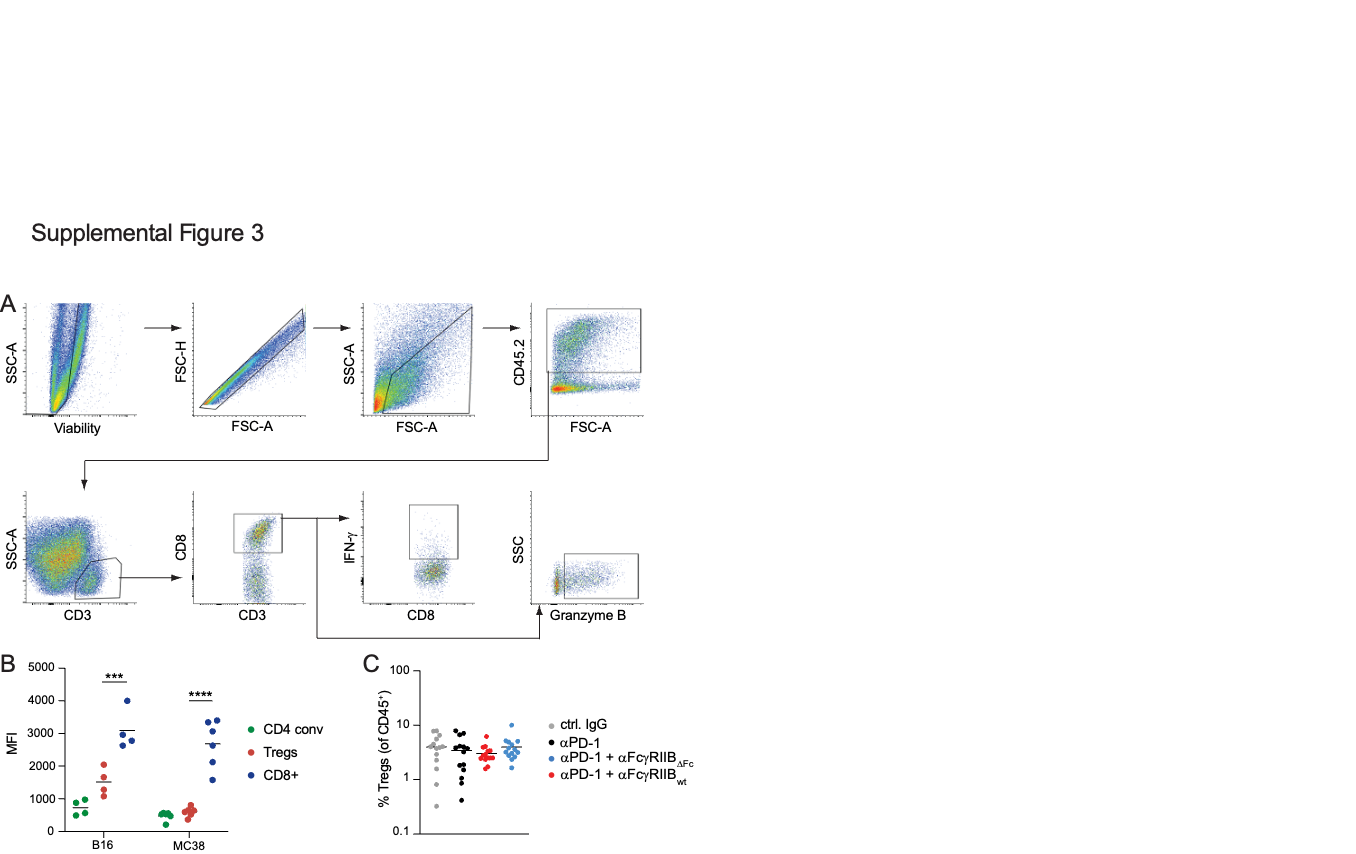


**Fig. S3.** αFcγRII_wt_ enhances αPD-1-induced antitumor activity in MC38 tumor-bearing mice. Related to Fig. 4C. **(A)** Flow cytometry gating strategy to assess intratumoral CD8^+^ T cells in MC38 tumor-bearing C57BL/6 mice, as shown in Fig. 4C.**(B)** PD-1 is expressed at higher levels on tumor-infiltrating CD8+ T cells taken from B16 or MC38 tumors, compared with Treg cells **(***** p<0.001, **** p<0.0001). **(C) α**PD-1 does not induce Treg depletion, and αFcγRIIB does not modulate Treg proportions in αPD-1-treated MC38 tumor-bearing C57BL/6 mice (pooled data from two experiments, *n*=14).
